# A Collection of Single-Domain Antibodies that Crowd Ricin Toxin’s Active Site

**DOI:** 10.3390/antib7040045

**Published:** 2018-12-17

**Authors:** Siva Krishna Angalakurthi, David J. Vance, Yinghui Rong, Chi My Thi Nguyen, Michael J. Rudolph, David Volkin, C. Russell Middaugh, David D. Weis, Nicholas J. Mantis

**Affiliations:** 1 Department of Pharmaceutical Chemistry and Macromolecule and Vaccine Stabilization Center, University of Kansas, Lawrence, KS 660451, USA; s214a774@ku.edu (S.K.A.); volkin@ku.edu (D.V.); middaugh@ku.edu (C.R.M.); 2 Division of Infectious Diseases, Wadsworth Center, New York State Department of Health, Albany, NY 12208, USA; david.vance@health.ny.gov (D.J.V.); yinghui.rong@health.ny.gov (Y.R.); 3 New York Structural Biology Center (NYSBC), New York, NY 10027, USA; ntchimy@gmail.com (C.M.T.N.); mrudolph@nysbc.org (M.J.R.); 4 Department of Chemistry and Ralph Adams Institute for Bioanalytical Chemistry, University of Kansas, Lawrence, KS 660451, USA; dweis@ku.edu

**Keywords:** toxin, antibody, camelid, vaccine, biodefense, hydrogen exchange-mass spectrometry

## Abstract

In this report, we used hydrogen exchange-mass spectrometry (HX-MS) to identify the epitopes recognized by 21 single-domain camelid antibodies (V_H_Hs) directed against the ribosome-inactivating subunit (RTA) of ricin toxin, a biothreat agent of concern to military and public health authorities. The V_H_Hs, which derive from 11 different B-cell lineages, were binned together based on competition ELISAs with IB2, a monoclonal antibody that defines a toxin-neutralizing hotspot (“cluster 3”) located in close proximity to RTA’s active site. HX-MS analysis revealed that the 21 V_H_Hs recognized four distinct epitope subclusters (3.1–3.4). Sixteen of the 21 V_H_Hs grouped within subcluster 3.1 and engage RTA α-helices C and G. Three V_H_Hs grouped within subcluster 3.2, encompassing α-helices C and G, plus α-helix B. The single V_H_H in subcluster 3.3 engaged RTA α-helices B and G, while the epitope of the sole V_H_H defining subcluster 3.4 encompassed α-helices C and E, and β-strand h. Modeling these epitopes on the surface of RTA predicts that the 20 V_H_Hs within subclusters 3.1–3.3 physically occlude RTA’s active site cleft, while the single antibody in subcluster 3.4 associates on the active site’s upper rim.

## 1. Introduction

**Figure 1:**
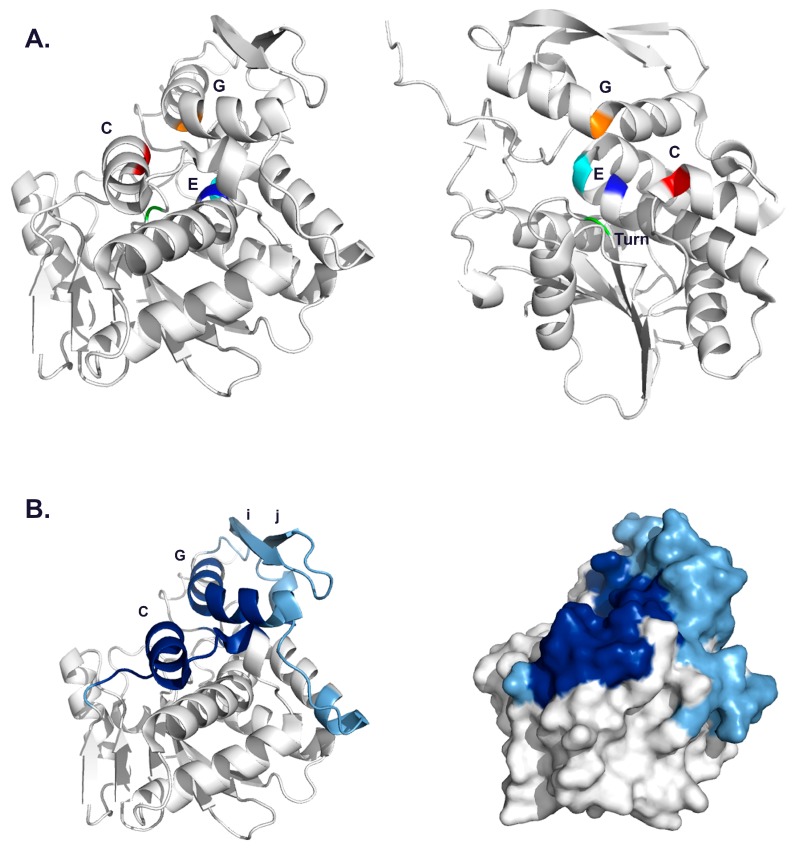
Structure of enzymatic subunit (RTA) with active site residues and IB2’s epitope highlighted. (**A**) The residues that constitute the RTA’s active site are in α-helices C, G and E, and a loop between β-strands e and f. The following residues are colored: Tyr80 (green); Tyr123 (red); Glu 177 (blue); Arg 180 (cyan) Trp211 (orange). (**B**) IB2’s epitope on RiVax adapted from previous publication [11]. The color shading corresponds to strong (deep blue) and intermediate (light blue) protection. No significant interaction is colored gray.

Ricin is a member of the ribosome-inactivating protein (RIP) family of toxins and classified as a biothreat agent due to its high potential to induce morbidity and mortality after inhalation [1,2,3]. The toxin is a ~65 kDa heterodimeric glycoprotein from the castor bean plant (*Ricinus communis*) consisting of a binding subunit (RTB) and an enzymatic subunit (RTA). RTB is a galactose/N-acetyl galactosamine (Gal/GalNAc)-specific lectin that promotes toxin attachment and entry into mammalian cells [4]. RTA is an RNA N-glycosidase (EC 3.2.2.22) that depurinates a conserved adenosine within the sarcin-ricin loop (SRL) of 28S rRNA, thereby stalling ribosome translocation [5,6]. At the structural level, RTA is a globular protein with a total of 10 β-strands (A–J) and seven α-helices (A–G). RTA folds into three distinct domains: domain 1 (residues 1–117) is dominated by a six-stranded β-sheet, domain 2 (residues 118–210), by five α-helices, and domain 3 (residues 211–267), which interfaces with RTB through hydrophobic interactions and a single disulfide bond [7,8]. RTA’s active site constitutes a shallow pocket formed at the interface of the three domains [8,9]. Active site residues include Tyr80, Tyr123, Glu177, Arg180, and Trp211 (Figure 1A) [10]. 

Inhalation of ricin results in severe lung inflammation characterized by an influx of neutrophils, alveolar edema, and hemorrhage, presumably initiated by the intoxication of alveolar macrophages and lung epithelial cells [1,12,13]. Non-human primates (NHPs) exposed to ricin by aerosol succumb to the effects of the toxin within 24–52 h [12,14]. At the present time, medical intervention following ricin exposure is strictly supportive [15]. However, vaccination strategies have shown great promise in affording complete or near complete protection against ricin intoxicosis in mice and NHPs [16]. For example, intramuscular administration of RiVax, a non-toxic thermostabilized recombinant RTA-based subunit vaccine adjuvanted with aluminum salts, to Rhesus macaques was sufficient to confer immunity to a lethal dose (LD) ricin challenge delivered by aerosol [14]. In vivo neutralization of ricin toxin following vaccination is associated with onset of anti-RTA IgG antibodies in serum and bronchoalveolar lavage (BAL) fluid [13,14,17]. 

Monoclonal (mAb) and polyclonal (pAb) antibody responses in mice, rabbits, and NHPs elicited by RiVax vaccination are directed against four spatially distinct immunodominant regions on RTA, which we refer to as epitope clusters 1–4 [11,18,19,20,21,22,23]. A combination of competition ELISAs, X-ray crystallography, and hydrogen exchange-mass spectrometry (HX-MS) has revealed key secondary elements associated with each cluster. Cluster 1 encompasses RTA’s β-strand h (residues 113–117) and α-helix B (94–107), a protruding immunodominant secondary structure element previously known to be a target of potent toxin-neutralizing antibodies [24]. Cluster 2 consists of two subclusters: one involving α-helix A (14–24) and α-helices F–G (184–207) and the other encompassing β-strands d-e (62–69) and parts of α-helices D–E (154–164). Cluster 3 involves α-helices C (121–135) and G (207–217) near RTA’s active site, while Cluster 4 is proposed to form a diagonal sash from the front to back of RTA spanning β-strands b, c, and d (35–59). Our long-term goal is to generate a comprehensive molecular B-cell epitope map of each of these clusters and define the specific antibody-contact points on RTA that render the toxin inactive. Such information will be invaluable in efforts to deconvolute the complex human antibody response profile to ricin toxin and RiVax [25].

While much has been learned about clusters 1 and 2 over recent years, comparatively little is known about cluster 3, as it is defined by only a single mAb called IB2 [11]. IB2 was first identified as a toxin-neutralizing mouse mAb that, in competition ELISAs, proved to be distinct from other mAbs in our collection at the time [18,26]. IB2 can passively protect mice against a 5 × LD_50_ ricin challenge by injection, indicating it has neutralizing activity in vivo, and must, by definition, interact with an important element on ricin toxin. As noted above, we recently demonstrated by HX-MS analysis that IB2 recognizes an epitope involving RTA’s α-helix C (residues 121–135) and α-helix G (residues 207–217), which is not only in close proximity to RTA’s active site but includes two active site residues, Tyr123 and Trp211 (Figure 1B). However, efforts to interrogate cluster 3 in more detail have been hindered by the absence other cluster 3-specific mAbs. Indeed, recent screens of B-cell hybridomas derived from RiVax and ricin toxoid immunized mice failed to identify additional cluster 3 antibodies [27]. 

Whereas isolation of additional IB2-like mouse mAbs has not been fruitful, we did recently identify 21 unique heavy chain-only single-domain camelid antibodies (V_H_Hs) that are competed by IB2 for binding to ricin toxin (D. Vance, C. Shoemaker, N. Mantis, manuscript in preparation) [23]. We wished to characterize these V_H_Hs in detail with respect to their binding affinities, epitopes, and capacities to neutralize ricin. In this report, we localized by HX-MS the epitopes of all 21 of these V_H_Hs. We found that the 21 V_H_Hs fall within one of four distinct but overlapping subclusters (3.1–3.4) that share at least one secondary element contacted by IB2. Only two of the 21 V_H_Hs, V6D4 and V1D3, have appreciable toxin-neutralizing activity (TNA), which we speculate is due to their epitope specificity along with strong binding affinity to toxin. This work furthers our overall goal of constructing a complete B-cell epitope map of ricin toxin.

## 2. Materials and Methods

### 2.1. RiVax and V_H_H Production

RiVax was expressed and purified from *E. coli*, as described [28]. Please note that RiVax differs from native RTA at two positions, which render the enzyme inactive: there is an Ala at position 80 substituted for Tyr, and a Met at position 76 in place of Val [29]. RiVax also lacks high mannose residues normally found on RTA, due to the fact that RiVax is expressed in *E. coli*. In addition, the RiVax used here has the addition of an Ala at the N-terminus, which we denoted as residue 0 for simplicity. V_H_Hs were expressed in *E. coli* as either thioredoxin- and E-tagged constructs or tag-free variants [22]. 

### 2.2. Competition ELISA

NUNC microtiter plates (Fisher Scientific, Hampton, NH) were coated with competitor mAbs (1 µg/mL in Phosphate Buffered Saline (PBS)) overnight at 4 °C and then blocked for 2 h with 2% goat serum (Gibco, Gaithersburg, MD, USA) in 0.1% PBST. Ricin (1 µg/mL) (Vector Labs, Burlingame, CA, USA) was then captured by the mAbs and probed with V_H_H analytes at 330 nM. Bound V_H_Hs were detected with an anti-E-tag-HRP secondary antibody (Bethyl Labs, Montgomery, TX, USA) and developed with SureBlue 3,3′,5,5′-tetramethylbenzidine (TMB) substrate (SeraCare, Milford, MA, USA). After quenching with 1 M phosphoric acid (Sigma Aldrich, Carlsbad, CA, USA), absorbance was read at 450 nm on a VersaMax microplate reader (Molecular Devices, Sunnyvale, CA, USA). % inhibition was calculated by comparing absorbance of captured V_H_Hs on each mAb-ricin complex with that of the absorbance of each V_H_H captured onto SylH3-ricin, where SylH3 is an anti-RTB mAb that does not interfere with the binding of any V_H_Hs to RTA’s cluster 3.

### 2.3. Vero Cell Cytotoxicity Assay

Vero cells were detached from culture dishes with trypsin (Gibco), seeded into white 96-well cell culture treated plates (Fisher Scientific) (100 uL per well, 5 × 10^4^ cells/mL) and allowed to adhere overnight. The cells were then treated with Dulbecco’s Modified Eagle Medium (DMEM) alone, ricin alone (10 ng/mL), or a mixture of ricin with V_H_Hs at five-fold dilutions. After 2 h at 37 °C, the culture medium was changed, and the cells were incubated at 37 °C for ~48 h. Viability was assessed using CellTiter-GLO (Promega, Madison, WI, USA). All treatments were performed in triplicate and repeated at least three times.

### 2.4. Affinity Determinations

V_H_H association and dissociation rates were determined by SPR using a ProteOn XPR36 system (Bio-Rad Inc., Hercules, CA, USA). Ricin was immobilized on a general layer compact (GLC) chip (Bio-Rad Inc.) equilibrated in PBS-0.005% Tween running buffer at a flow rate of 30 µL/min. Following EDAC [N-ethyl-N=-(3-dimethylaminopropyl) carbodiimide hydrochloride] (200 mM)–sulfo-NHS (N-hydroxysulfosuccinimide) (50 mM) activation (3 min), ricin was diluted in 10 mM sodium acetate (pH 5.0) at either 4 µg/mL or 2 µg/mL and coupled for 2 min. A third vertical channel received only acetate buffer and served as a reference channel. The surfaces were deactivated using 1 M ethanolamine for 5 min. A ProteOn array system multichannel module (MCM) was rotated to the horizontal orientation for affinity determination experiments. Each V_H_H was serially diluted in running buffer and then injected at 50 µL/min for 180 s, followed by 1 to 3 h of dissociation. After each experiment, the chip was regenerated with 10 mM glycine (pH 1.5) at 100 µL/min for 18 s, until the response unit (RU) values had returned to baseline. All kinetic experiments were performed at 25 °C. Kinetic constants for the antibody/ricin interactions were obtained with ProteOn Manager software 3.1.0 (Bio-Rad Inc.) using the Langmuir fit model.

### 2.5. HX-MS

HX-MS experiments for epitope mapping were conducted essentially as described previously [11]. Briefly, a H/DX PAL™ robotic system (LEAP Technologies, Morrisville, NC, USA) was used for sample preparation, mixing and injection. For the free RiVax, 4 µL of 20 µM RiVax stock solution was incubated with 36 µL of deuterated buffer (10 mM sodium phosphate, 150 mM sodium chloride, pD 7.4). For the bound states, the stock solution had a final concentration of 20 µM RiVax and 40 µM V_H_H resulting in 1:2 molar ratio of RiVax:V_H_H. Four µL of the stock was incubated with 36 µL of deuterated buffer. Samples were incubated at 25 °C for five HX times between 13 s and 24 h and subsequently quenched using 200 mM phosphate-4 M guanidine hydrochloride solution (pH 2.5) held at 0 °C. The quenched samples were then injected onto an immobilized pepsin column where proteolysis occurs overlapping peptides from RiVax. The peptides were desalted using a C18 trap and separated using a segmented gradient with water/acetonitrile/0.1% formic acid on a C18 column (Zorbax 300SB-C18 2.1 × 50 mm, 1.8 μm particle diameter, Agilent, Santa Clara, CA, USA). The entire liquid chromatography system (immobilized pepsin column, C18 trap and a C18 RP-UHPLC column) was kept in a refrigerated cabinet that is maintained at 0 °C to minimize back exchange. Nevertheless, the first two residues in a peptide generally undergo rapid back exchange [30]. RiVax peptides were analyzed by an QTOF mass analyzer (model 6530, Agilent Technologies, Santa Clara, CA, USA) for their increase in mass i.e., for deuterium uptake. All HX-MS measurements were based on triplicate independent HX reactions of each labeling time.

### 2.6. Data Analysis

The HX-MS data processing was carried out using HDExaminer (version 2.3, Sierra Analytics, Modesto, CA, USA). A total of 138 peptides (Table S1) that cover the entire sequence of RiVax were analyzed. For each peptide, the magnitude of protection from each HX time was averaged and normalized to its peptide length to obtain a ΔHX value, ΔHX = HX_bound_ − HX_bound_, as described previously [11]. The propagated standard error in delta HX was estimated as described in [31]. The magnitudes of delta HX values of overlapping peptides that span the entire RiVax are then classified using K-means clustering into three categories and were colored as follows: strong protection, intermediate protection, no significant protection. For visualization, the HX-MS results were mapped onto the crystal structure of RiVax (PDB: 3SRP) [32] using PyMoL (The PyMOL Molecular Graphics System; Schrodinger LLC, San Diego, CA, USA). For better visualization purpose, only overlapping peptides that fall in strong and intermediate protection category are colored. 

## 3. Results

### 3.1. Identification and Characterization of Cluster 3 V_H_Hs

**Figure 2:**
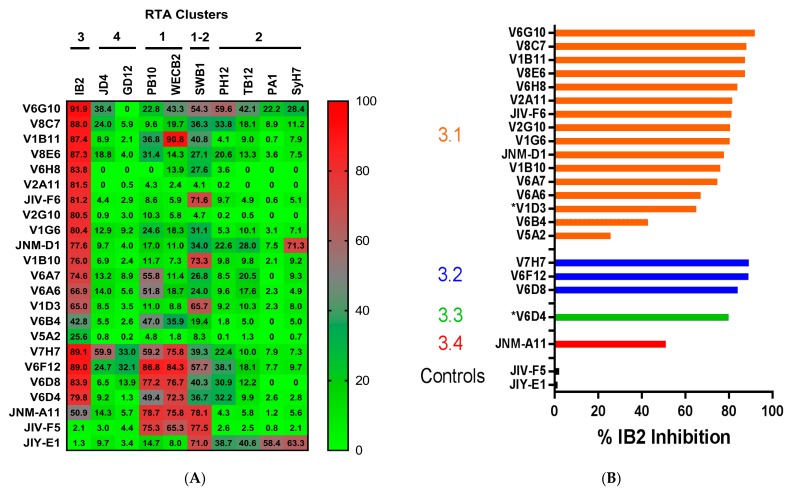
V_H_H binning by competition ELISA. (**A**) Ricin was captured on microtiter plates by anti-RTA mAbs (indicated along the top panel) or an anti-RTB mAb (SylH3) as a control. The plates were then probed with individual V_H_Hs as indicated on the left most column and detected with a secondary E-tag antibody. Binding inhibition was calculated as 100 − (100 × (A_mAb-Ricin_/A_SylH3-Ricin_)) where interference by SylH3 was assumed to be negligible. The colored scale bar on far right indicates % inhibition. V_H_Hs JIV-F5 and JIY-E1 were used as controls, since they are known to bind epitopes on RTA outside of IB2’s footprint. (**B**) The IB2 values from panel A are re-plotted for clarity to compare relative IB2 inhibition values and color coded based on subcluster designations described later in the manuscript. The two V_H_Hs with toxin-neutralizing activity are denoted with an *.

Using a variety of screening strategies that are described in detail in separate manuscripts (D. Vance, J. Tremblay, C. Shoemaker, N. Mantis, manuscript in preparation) [23], we identified from different phage-displayed alpaca single chain libraries a total of 21 V_H_Hs whose binding to ricin toxin was partially or completely inhibited by IB2 in a capture ELISA (Figure 2). The competitive ELISA was designed such that IB2 was immobilized on microtiter plates and then allowed to capture ricin in solution. The plates were washed to remove unbound ricin and then probed with query V_H_Hs, as described in the figure legend and Materials and Methods. The DNA sequences and mAb competition profiles of ten of the V_H_Hs were reported in a recent study, although only two (JNM-D1 and V1B11) HX-MS epitopes were described [23].

To further differentiate among the 21 V_H_Hs, they were subjected to a more comprehensive competition array using a panel of nine additional RTA-specific mAbs representing cluster 1 (PB10, WECB2), cluster 1–2 (SWB1), cluster 2 (PH12, TB12, PA1, SyH7), and cluster IV (JD4, GD12) [11]. The competition ELISA revealed a wide range of profiles (Figure 2), indicating the 21 V_H_Hs, as a whole, represent a diversity of epitopes on RTA. Indeed, the predicted CDR3 amino acid sequences of the 21 V_H_Hs suggest they represent at least 11 different B-cell lineages: five unique V_H_Hs and 16 others that fell into one of six sequence families (Table 1; Figure S1). 

The binding kinetics of each V_H_H for ricin holotoxin was determined by surface plasmon resonance (SPR). Twelve of the 21 V_H_Hs had dissociation constants (*K_d_*) of greater than 1 nM, while the remaining nine had dissociation constants ranging from 0.2–1 nM (Table 2; Figure S2). The V_H_Hs were also tested for ricin TNA in a Vero cell assay. Only two V_H_Hs, V6D4 (IC_50_, 200 nM) and V1D3 (IC_50_, 80 nM), had demonstrable TNA (Figure S3). Neutralizing activity was not solely a function of binding affinity, as several V_H_Hs with K_D_s comparable to V6D4 and V1D3 lacked detectable neutralizing activity. For that reason, we sought to localize the epitopes on RTA recognized by each of the 21 V_H_Hs with the expectation that such information would offer insight into the basis of toxin-neutralizing activity. 

### 3.2. V_H_H Epitope Mapping by HX-MS

**Figure 3:**
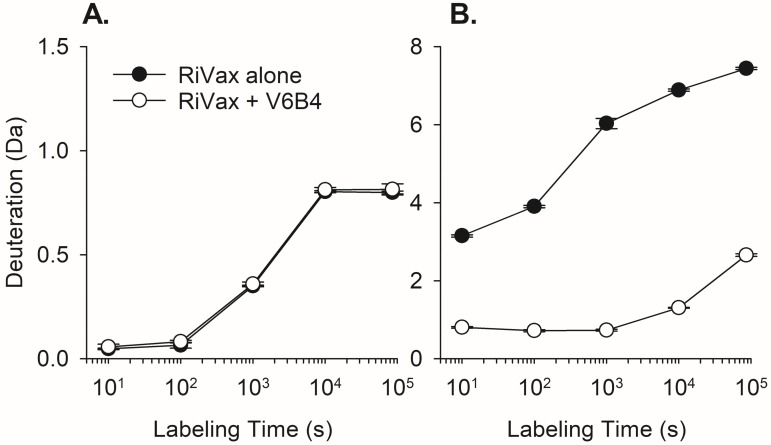
Hydrogen exchange (HX) kinetics of two representative RiVax peptides in presence of V6B4. Hydrogen deuterium exchange kinetics of two representative RiVax peptides in presence of V6B4. (**A**) Peptide 14 (56–59), where the HX rate was not affected by association with V6B4. (**B**) Peptide 94 (205–217) where the rate of HX was substantially slowed by V6B4. Significance limit for HX differences was defined as described in the Experimental section.

We have previously used HX-MS to localize more than two dozen V_H_H and mAb epitopes on RTA or on RiVax, an attenuated recombinant RTA subunit vaccine antigen with point mutations at positions V76 and Y80 [11,23,27,31,33]. We used RiVax in place of RTA because it is non-toxic to humans and therefore poses no hazard to research staff. RiVax also assumes a tertiary structure essentially identical to RTA [32]. Therefore, HX-MS was performed with RiVax in the presence of two-fold molar excess of each of the cluster 3 V_H_Hs at five exchange times between 13 s and 24 h. Epitope assignment was based on reduced (slower) HX exchange for peptides in the presence of a V_H_H, as compared to RiVax alone. For example, in the presence of V6B4, the HX rate in the peptide corresponding to RiVax residues 57–60 was unaltered, whereas there was much slower exchange observed for the peptide corresponding to residues 206–218 (Figure 3). While reduced hydrogen exchange is generally attributed to direct antibody-protein interaction, we cannot necessarily exclude possible allosteric effects that may occur upon antibody engagement, especially when reduced exchange is observed at a distance not consistent with being part of a core epitope [11]. 

### 3.3. Identification of Epitope Subclusters

The results of epitope mapping studies revealed that the Cluster 3 V_H_Hs grouped within four subclusters, referred to as 3.1–3.4 (Table 3; Table S2;). Subcluster 3.1 involves contact with RiVax α-helices C and G, a profile very similar to mAb IB2. Subcluster 3.2 encompasses α-helices B, C and G, while subcluster 3.3 covers α-helices B and G, but not α-helix C. Finally, subcluster 3.4 encompasses α-helices C and E, but not G. Each of these subclusters is now described in more detail. 

**Figure 4:**
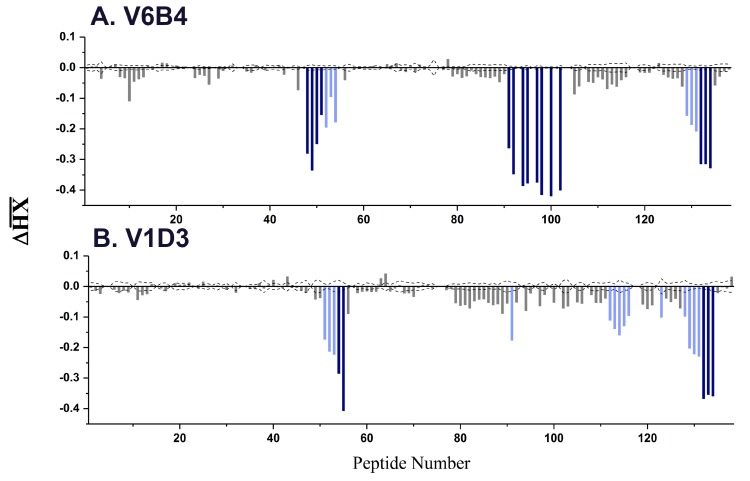
HX-MS analysis of RiVax bound to two V_H_Hs in subcluster 3.1. The $\Delta \bar{\mathrm{HX}}$ values for each RiVax peptide are shown for V_H_Hs (**A**) V6B4 and (**B**) V1D3. The $\Delta \bar{\mathrm{HX}}$ values are clustered using k-means clustering into three categories: strong (deep blue), intermediate (light blue) or no significant protection (gray). The dotted lines represent “3σ” confidence intervals for statistically significant changes in hydrogen exchange.

**Figure 5:**
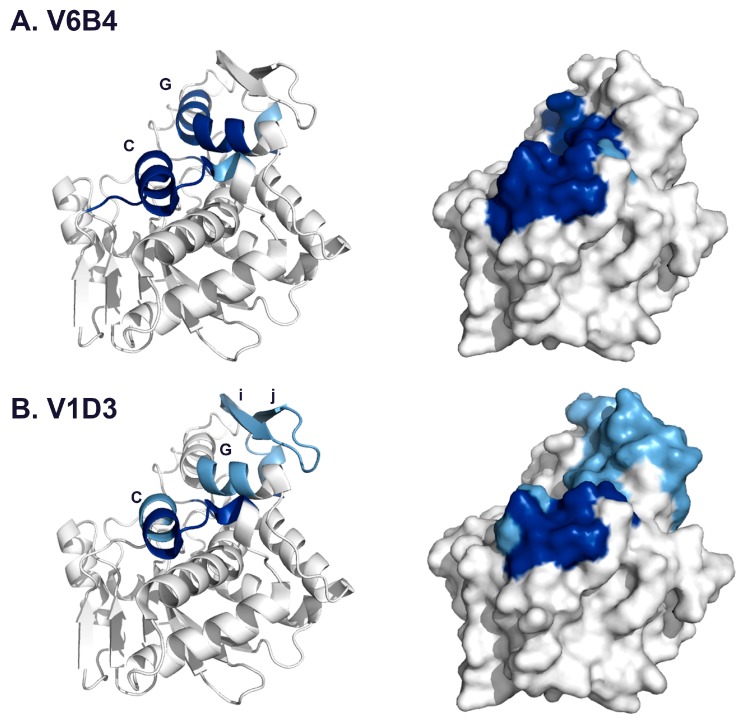
Epitope localization for two subcluster 3.1 V_H_Hs. HX protection categories shown in Figure 4 were mapped onto the crystal structure of RiVax for (**A**) V6B4 and (**B**) V1D3. The most relevant secondary structure elements, α-helices C and G and β-strands i and j, are labeled. The color shading corresponds to strong (deep blue), intermediate (light blue) or no significant protection (gray), as represented in Figure 4.

Subcluster 3.1: Sixteen of the 21 V_H_Hs shared an HX-MS profile involving contact with α-helices C and G, which we refer to as subcluster 3.1 (Table 3; Table S2; Figures S4 and S5). While the HX-MS profiles of the V_H_Hs within 3.1 were qualitatively similar, there were quantitative differences that may be significant in terms of neutralizing activity. For example, V1D3, one of the two V_H_Hs with toxin-neutralizing activity, had a binding pattern virtually identical to IB2 in that it strongly protected α-helix C (peptides 54–55, residues 127–135) and the C-terminus region (peptides 132–134, residues 249–255) (Figure 4 and Figure 5). Moreover, V1D3 demonstrated intermediate protection of α-helix G (peptide 91, residues 205–210), as well as strands i and j (peptides 112–116, residues 226–243). In contrast, V6B4, an antibody without toxin-neutralizing activity, strongly protected RiVax residues 119 to 133 (peptides 48–51), corresponding to α-helix C, and residues 205–217 (peptides 91 to 102), corresponding to α-helix G (Figure 4 and Figure 5). However, V6B4 differed from V1D3 in three respects. First, V6B4 had stronger protection of α-helix G than C, as compared to V1D3. Second, V1D3 interacted with β-strands i and j, while V6B4 did not, possibility indicating that V1D3 overall contact interface with RiVax is larger than V6B4’s. Finally, the patterns of protection in α-helix C are distinct. In case of V6B4, the entirety of α-helix C is strongly protected, while in the case of V1D3 it is only the C-terminal end that is strongly protected (Figure 4 and Figure 5). V1D3 also caused intermediate protection in the N-terminal end of helix G, while V6B4 protected all of helix G. It is unclear if these differences in α-helix C and α-helix G protection explain V1D3’s TNA. 

The competition ELISA with the panel of RTA-specific mAbs revealed additional degrees of difference among the 16 V_H_Hs in subcluster 3.1 (Figure 2). Not only was there a clear gradation of competition with IB2 (range 25–90%), but there were marked disparities with other mAbs. For example, V1B11 is a potent inhibitor of WECB2, V1D3 stood out because of competition with SWB1, while JNM-D1 competes strongly with SyH7. Because the footprints of all 10 anti-RTA mAbs have been defined, we can infer from the various inhibition profiles how different V_H_Hs engage RTA. Thus, looking directly at the RTA active site, with RTB oriented on the bottom, we predict that V1B11 likely approaches RTA from the top down, V1D3 likely from top left, and JNM-D1 likely from bottom left. 

**Figure 6:**
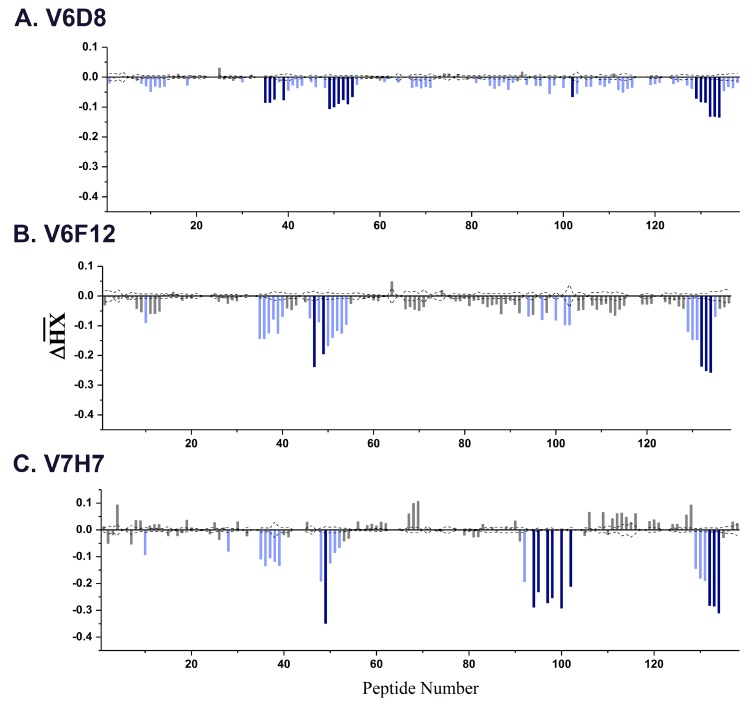
HX-MS analysis of RiVax bound to two V_H_Hs in subcluster 3.2. The $\Delta \bar{\mathrm{HX}}$ values for each RiVax peptide are shown for V_H_Hs (**A**) V6D8 (**B**) V6F12 and (**C**) V7H7. The $\Delta \bar{\mathrm{HX}}$ values are clustered using k-means clustering into three categories: strong (deep blue), intermediate (light blue) or no significant protection (gray). The dotted lines represent “3σ” confidence intervals for statistically significant changes in hydrogen exchange.

**Figure 7:**
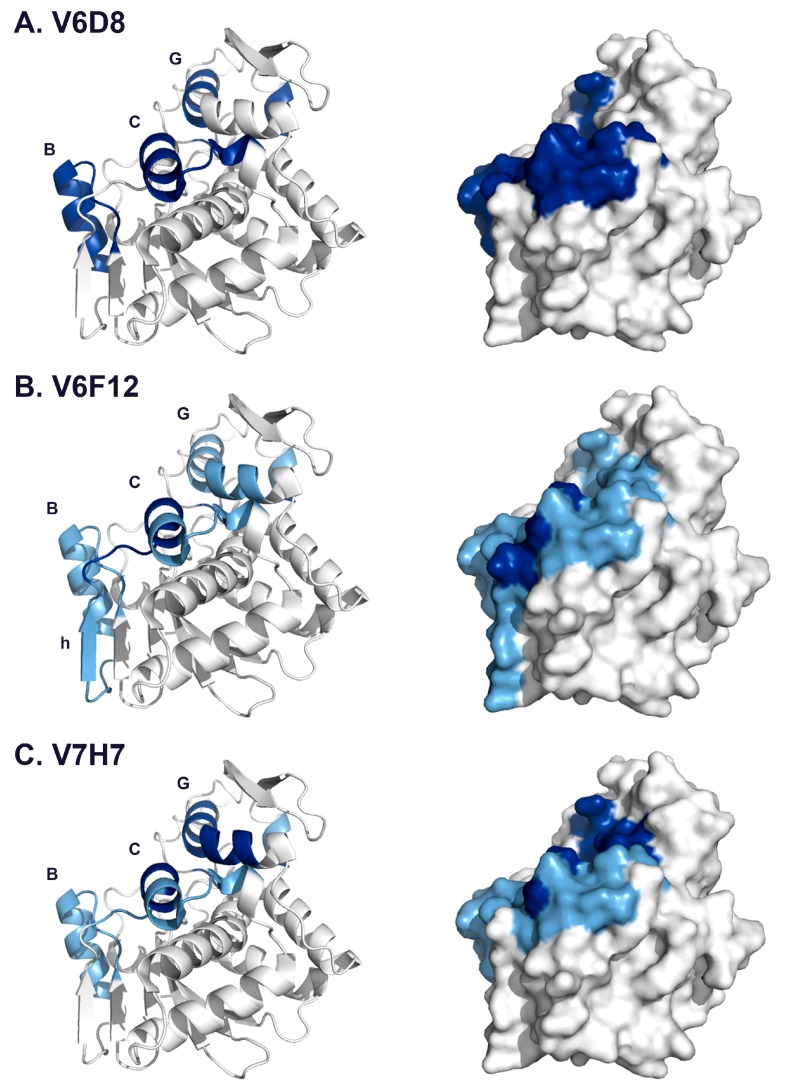
Epitope locations of V6D8, V6F12 and V7H7 on RiVax. HX protection categories shown in Figure 6 were mapped onto the structure of RiVax for (**A**) V6D8 (**B**) V6F12 and (**C**) V7H7. Secondary structure elements including α-helices B, C and G and β-strand h are labeled. Intermediate protection by V6D8 is spread over much of RiVax’s surface and the magnitudes of protection are low. Therefore, only strongly protected elements are mapped onto the crystal structure of RiVax. The color shading corresponds to strong (deep blue) or no significant protection (gray).

Subcluster 3.2: Three V_H_Hs, V6D8, V6F12 and V7H7, were grouped within subcluster 3.2 based on a common HX-MS profile encompassing α-helices B, C and G (Figure 6 and Figure 7). For example, V6D8 strongly protected α-helices B (residues 92–107; peptides 35–39), C (residues 123–135; peptides 49–54), G (residues 211–217; peptides 102) and a short region near the C-terminus of α-helix G (residues 247–255, peptides 129–134). V6F12 shared a binding profile with V6D8, which was not surprising since the two V_H_Hs are likely from the same B-cell lineage (Table 1; Figure S1). Although the protection profiles of V6D8, V6F12, and V7H7 were qualitatively similar, and all three V_H_Hs were competed by IB2 to a similar degree, the magnitudes of protection in the three secondary structural features were distinct. V6D8 and V6F12 interacted primarily with α-helices B and C, and secondarily with α-helix G. V7H7, by contrast, primarily protected several overlapping peptides in α-helix G, and secondarily protected α-helices B and C (except for one peptide in α-helix C). Finally, HX-MS indicated that V6D8, V6F12, and V7H7 each contact α-helix B, which has been postulated as being a neutralizing hotspot on RTA [22]. However, none of the V_H_Hs within this subcluster had any detectable TNA, possibly because their binding affinities do not achieve a minimum threshold required to inactivate ricin. Other previously described V_H_Hs that engage α-helix B and have potent toxin-neutralizing activities each have binding affinities of less than 200 pM, including JIV-F5 (19 pM), JIY-E5 (191 pM), and JPF-A9 (102 pM) [21,22,33]. This contrasts with V7H7, the strongest binder in subcluster 3.2, which has a binding affinity of ~500 pM.

**Figure 8:**
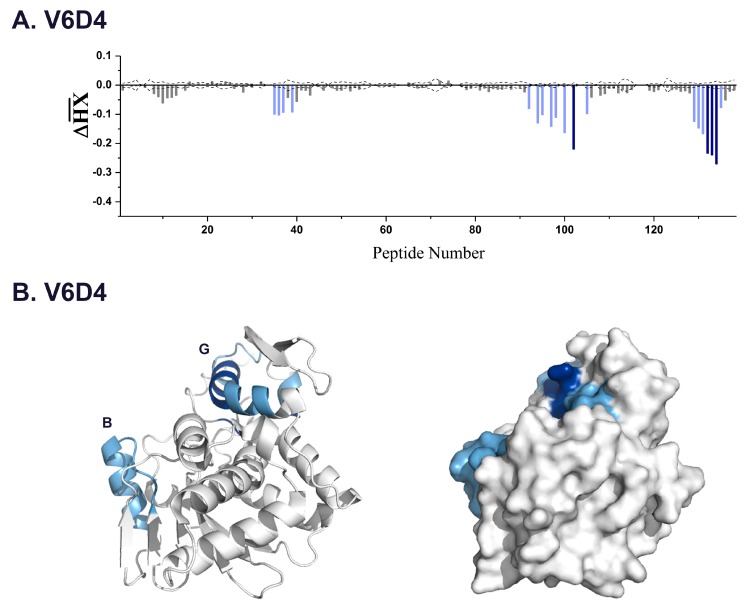
Epitope mapping of V6D4 from subcluster 3.3. (**A**) Relative levels of protection of RiVax peptides by V6D4 as defined by HX-MS. The color shading corresponds to strong (deep blue), intermediate (light blue) or no significant protection (gray), as represented in Figure 4. (**B**) The HX protection categories, as shown in panel A, were mapped onto the crystal structure of RiVax. Secondary structures α-helices B and G are labeled.

Subcluster 3.3: The third subcluster is populated by V6D4, which had weak toxin-neutralizing activity (IC_50_ ~200 nM) in the Vero cell cytotoxicity assay. HX-MS analysis demonstrated strong protection of α-helix G (peptide 102, residues 211–217) and intermediate protection of α-helix B (peptides 35–39, residues 92–107). V6D4 also protected a short region in the C-terminus of RiVax, but not α-helix C itself (Figure 8). Whether V6D4’s neutralizing activity is a result of contact with α-helix B is unclear, though its high affinity for ricin (*K_d_* = 222 pM) may put it above any relevant affinity threshold.

**Figure 9:**
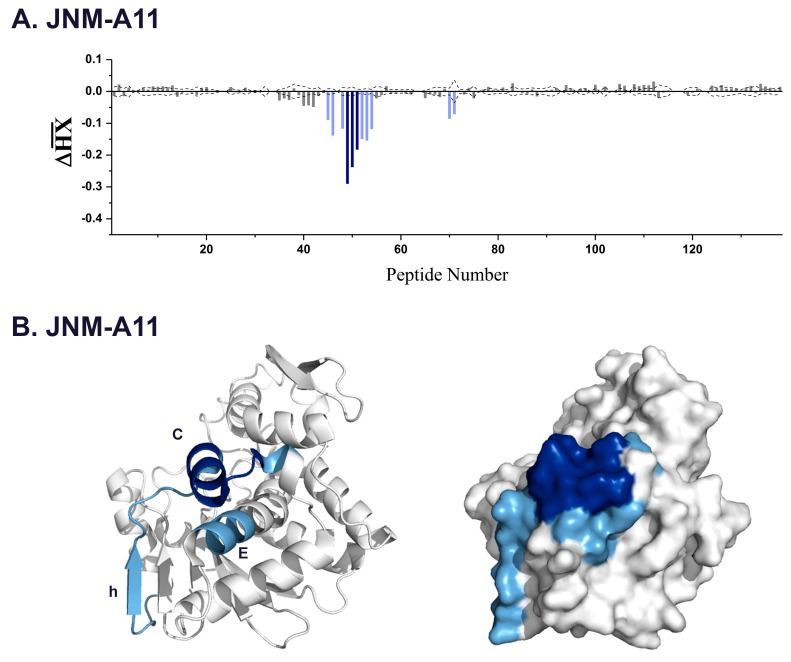
Epitope mapping of JNM-A11 from cluster 3.4. (**A**) Relative levels of protection of RiVax peptides by JNM-A11, as defined by HX-MS. The color shading corresponds to strong (deep blue), intermediate (light blue) or no significant protection (gray), as represented in Figure 4. (**B**) The HX protection categories, as shown in panel a, are mapped onto the crystal structure of RiVax. Secondary structures α-helices C and E and β-strand h are labeled.

Subcluster 3.4: The fourth subcluster is also populated by a single antibody, JNM-A11. JNM-A11 showed strong protection of residues in RTA’s α-helix C (peptides 49–51; residues 124–133) and intermediate protection of the N-terminal region of α-helix E (peptides 70 and 71; residues 162–168) and β-strand h (peptides 45 and 46; residues 108–122) (Figure 9). JNM-A11 did not protect α-helix G, which differentiates it from the 20 other V_H_Hs in cluster 3. JNM-A11’s competition profile against a panel of RTA-specific mAbs is consistent with results obtained by HX-MS. Namely, JNM-A11 competed with both Cluster 1 (PB10, WECB2) and cluster 1–2 (SWB1) mAbs (Figure 2A). Finally, JNM-A11 did not neutralize ricin, despite a strong binding affinity (*K_d_* = 212 pM). Since JNM-A11 appears to target α-helix C almost exclusively, we infer that contact with α-helix C alone is not sufficient to affect ricin function. 

## 4. Discussion

**Figure 10:**
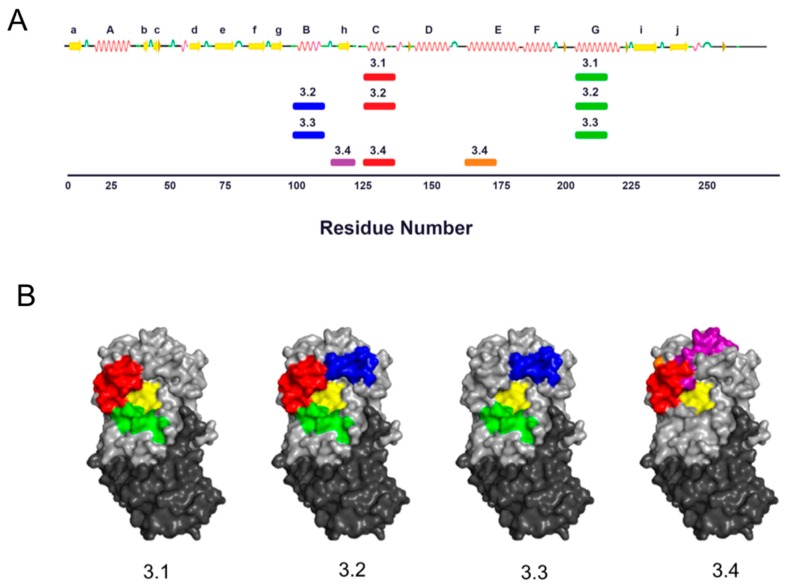
Visual representation subcluster 3 binding sites on ricin toxin. (**A**) Linear depiction of RTA with arrows denoting β-strand secondary structure and coils indicating α-helices, as per Protein Data Bank (PDB) format. Below, the colored bars denote epitope coverage for each of the V_H_H subclusters 3.1–3.4. The colors correspond to secondary structures highlighted in panel B. Horizontal line below refers to RTA amino acid residue number. (**B**) Surface representations of ricin (PDB 2AAI) using PyMol showing the regions of protection for each of the four subclusters (3.1–3.4). Colors are as follows: RTA, light gray; RTB, dark gray; active site, yellow; α-helix B, blue; α-helix C, red; α-helix G, green; α-helix E, orange; β-strand h, purple.

As part of our long-standing effort to generate a comprehensive B-cell epitope map of ricin toxin, we have characterized 21 unique V_H_Hs that share the common property of being within the shadow of IB2 based on competition ELISAs. IB2 is a toxin-neutralizing mAb that engages with α-helices C and G on RiVax and defines so-called epitope cluster 3 [11,18]. Cluster 3 is of interest because it encompasses the residues on RTA involved in ribosome inactivation [10,34,35]. The 21 V_H_Hs originated from different phage-displayed libraries, each generated from alpacas immunized with ricin toxin antigens, including RiVax (D. Vance, C. Shoemaker, N. Mantis, manuscript in preparation) [23]. As a result, of epitope mapping studies by HX-MS, the 21 V_H_Hs were further grouped into four distinct subclusters (3.1–3.4) based on their interactions with RiVax α-helix C and α-helix G, as well as other local secondary structures including α-helix B, α-helix E, and β-strand h (Figure 10). The fact that all 21 V_H_Hs engage RiVax via α-helix C and/or α-helix G explains the observed competition with IB2 by ELISA (Figure 2). However, we are unable to explain exactly why V1D3 and V6D4 are the only V_H_Hs within cluster 3 that have toxin-neutralizing activity, since other V_H_Hs have similar footprints on ricin and nearly identical binding affinities as V1D3 and V6D4 but are devoid of neutralizing activity. We can only speculate that neutralizing activity is due to specific residue contacts or combinations of contact that are not apparent by HX-MS epitope mapping methodologies (see below). 

RTA’s active site consists of a large solvent-exposed cleft on one face of the molecule [10,34,35,36,37]. Active site residues include Tyr80, Tyr123, Arg180, and Glu177, which are involved in stacking the purine ring of target adenosine moiety (Tyr80, Tyr123) and transition state stabilization (Glu177, Arg180). Viewing the active site pocket head on, α-helices C (121–135) and G (207–217) would be located at 10 o’clock and 7 o’clock, respectively (Figure 10). Thus, antibodies in subclusters 3.1, 3.2 and 3.3 would be expected to physically occlude (straddle) or even occupy the active site pocket, whereas the single antibody (JNM-A11) in subcluster 3.4 is probably associated with upper rim (11 o’clock) of the active site (Figure 10). To examine these possibilities, efforts are ongoing to solve the X-ray crystal structures of all 21 of these V_H_Hs in complex with RTA.

The current study also highlights both the advantages and shortcomings of HX-MS for use in B-cell epitope mapping. On the upside, the HX-MS pipeline proved to be robust and relatively high throughput due to the fact that we had already established a RiVax peptide map and baseline HX kinetics [11]. HX-MS was able to assess RiVax-V_H_H binding in solution and parse cluster 3 epitopes into four subclusters that we are currently compared to interaction sites observed by X-ray crystallography. On the other hand, HX-MS provides only peptide level resolution in terms of defining actual antibody contacts on the target antigen and cannot reveal subtle interactions that may ultimately be of consequence to toxin-neutralizing activity. As a case in point, we recently described two V_H_Hs (JPF-A9 and V8A7) with essentially identical HX-MS profiles but that differ in both binding affinity and toxin-neutralizing activity as a result of a single residue difference in CDR2 [33]. Coupling HX-MS with high density competition ELISAs and/or site-directed mutagenesis can significantly improve epitope definition [23,38,39,40]. The magnitude of HX protection will depend on the affinity and kinetics of binding. Lower affinity generally leads to weaker protection against HX, thereby making it more difficult to resolve the epitope from allosteric effects. However, in practice we have found that introduction of point mutations in V_H_Hs that led to ~10-fold differences in binding affinity (e.g., 0.4 to 4 nM) did not notably alter their HX profiles [31]. Since each epitope mapping data set is treated independently, our analysis still finds the most strongly protected regions. 

At this point in time, more than 30 alpaca B-cell epitopes and more than a dozen murine B-cell epitope on RTA have been reported [18,19,21,22,23,41,42,43,44]. The availability of this dense epitope map and a collection well characterized antibodies has already proven to have utility in terms of pre-clinical evaluation of RiVax and other candidate RTA-based vaccine antigens. In one instance the mAbs were used as tools in competition ELISAs to demonstrate epitope use within humans and non-human primates vaccinated with RiVax [14]. More recently, the mAbs were used to evaluate the integrity of key neutralizing epitopes on RiVax during long-term storage [25]. The 21 V_H_Hs described here focused around RTA’s active site now add to that growing list of critical reagents. 

## Figures and Tables

**Table 1:** V_H_H Families based on CDR3 similarity.

Family	Members
V1D3 *	JIV-F6, V1B10
V2A11	V6H8
V2G10	V1G6
V6D8	V6F12
V6A6	V6A7, V6G10, V8C7, V8E6
V6D4 *	V6B4

*, indicates V_H_Hs with toxin-neutralizing activity; The following V_H_Hs were not assigned to a family: JNM-A11, JNM-D1, V1B11, V5A2, V7H7.

**Table 2:** Cluster 3 V_H_H TNA and Binding Affinities.

V_H_H	Subcluster	IC_50_ (nM)	*K*_D_^a^ (nM)	*k*_on_^b^	*k*_off_^c^
V1D3	3.1	80	0.460	3.15 × 10^5^	1.45 × 10^−4^
V8C7		-	0.597	1.58 × 10^5^	9.40 × 10^−5^
V6B4		-	0.652	1.70 × 10^5^	1.11 × 10^−4^
V8E6		-	0.830	1.26 × 10^5^	1.04 × 10^−4^
V1B10		-	0.917	8.29 × 10^4^	7.60 × 10^−5^
V6A6		-	0.996	5.06 × 10^5^	5.04 × 10^−4^
V6H8		-	1.150	6.63 × 10^4^	7.66 × 10^−5^
V2G10		-	1.160	8.48 × 10^4^	9.84 × 10^−5^
JNM-D1		-	1.190	1.80 × 10^5^	2.15 × 10^−4^
V6G10		-	1.270	1.77 × 10^5^	2.24 × 10^−4^
V5A2		-	1.460	2.15 × 10^5^	3.14 × 10^−4^
V6A7		-	1.760	7.70 × 10^4^	1.36 × 10^−4^
V2A11		-	1.820	2.97 × 10^4^	5.41 × 10^−5^
JIV-F6		-	1.860	1.94 × 10^5^	3.61 × 10^−4^
V1G6		-	5.340	3.05 × 10^4^	1.63 × 10^−4^
V1B11		-	8.840	2.76 × 10^4^	2.44 × 10^−4^
V7H7	3.2	-	0.507	1.65 × 10^5^	8.36 × 10^−5^
V6D8		-	1.130	2.14 × 10^5^	2.41 × 10^−4^
V6F12		-	1.210	1.80 × 10^5^	2.17 × 10^−4^
V6D4	3.3	200	0.222	1.44 × 10^5^	3.21 × 10^−5^
JNM-A11	3.4	-	0.212	4.20 × 10^5^	8.91 × 10^−5^

^a^, determined by SPR with Langmuir fit model; ^b^, 1/Ms; ^c^, 1/s.

**Table 3:** Localization of epitopes on RiVax recognized by representative Cluster 3 V_H_Hs.

		Strong and Intermediate Protected Elements in RiVax ^a^		
V_H_H	Subcluster	Peptides	Residues	Structure(s)
V6B4	3.1	48–51	119–133	α-helix C
		91–102	205–217	α-helix G
		132–134	249–255	C-terminus
				
V1D3 *		54,55	127–135	α-helix C
		91	205–210	α-helix G
		112–116	226–243	β-strands i, j
		132–134	249–255	C-terminus
V6D8	3.2	35–39	92–107	α-helix B
		49–54	123–135	α-helix C
		102	211–217	α-helix G
		129–134	247–255	C-terminus
V6F12		35–40	92–107	α-helix B
		47,49	118–126	α-helix C
		94,97,100,102–103	205–217	α-helix G
		132–134	249–255	C-terminus
V7H7		35–39	92–107	α-helix B
		49	123–126	α-helix C
		94–95,97–98,100,102	205–217	α-helix G
		129–131	249–255	C-terminus
V6D4 *	3.3	35–37,39	92–107	α-helix B
		102	211–217	α-helix G
		132–134	249–255	C-terminus
JNM-A11	3.4	45,46	108–122	β-strand h
		49–51	124–133	α-helix C
		70,71	162–168	α-helix E

^a^, Peptides on RiVax are indicated in supplementary Table S1. *, indicates V_H_Hs with toxin-neutralizing activity. Underline indicates intermediate protection determined by HX-MS.

## Supplementary Materials

The following are available online at http://www.mdpi.com/2073-4468/7/4/45/s1, Table S1: RiVax peptic peptides, Table S2: HX-MS analysis of V_H_Hs in subcluster 3.1, Figure S1: Alignment of cluster 3 V_H_H families, Figure S2: Representative sensorgrams of Cluster 3 V_H_Hs, Figure S3: Representative toxin-neutralizing activities of cluster 3 V_H_Hs, Figure S4: HX-MS analysis of RiVax bound to V_H_Hs in subcluster 3.1, Figure S5: Epitope localization of subcluster 3.1 V_H_Hs on the surface of RiVax.

## References

[1] Gal Y., Mazor O., Falach R., Sapoznikov A., Kronman C., Sabo T. (2017). Treatments for pulmonary ricin intoxication: Current aspects and future prospects. Toxins (Basel).

[2] Griffiths G.D. (2011). Understanding ricin from a defensive viewpoint. Toxins (Basel).

[3] Reisler R.B., Smith L.A. (2012). The need for continued development of ricin countermeasures. Adv. Prev. Med..

[4] Rutenber E., Ready M., Robertus J.D. (1987). Structure and evolution of ricin b chain. Nature.

[5] Endo Y., Mitsui K., Motizuki M., Tsurugi K. (1987). The mechanism of action of ricin and related toxic lectins on eukaryotic ribosomes. The site and the characteristics of the modification in 28 s ribosomal rna caused by the toxins. J. Biol. Chem..

[6] Endo Y., Tsurugi K. (1987). Rna n-glycosidase activity of ricin a-chain. Mechanism of action of the toxic lectin ricin on eukaryotic ribosomes. J. Biol. Chem..

[7] Montfort W., Villafranca J.E., Monzingo A.F., Ernst S.R., Katzin B., Rutenber E., Xuong N.H., Hamlin R., Robertus J.D. (1987). The three-dimensional structure of ricin at 2.8 a. J. Biol. Chem..

[8] Rutenber E., Katzin B.J., Ernst S., Collins E.J., Mlsna D., Ready M.P., Robertus J.D. (1991). Crystallographic refinement of ricin to 2.5 a. Proteins.

[9] Katzin B.J., Collins E.J., Robertus J.D. (1991). Structure of ricin a-chain at 2.5 A. Proteins.

[10] Monzingo A.F., Robertus J.D. (1992). X-ray analysis of substrate analogs in the ricin a-chain active site. J. Mol. Biol..

[11] Toth R.T.I., Angalakurthi S.K., Van Slyke G., Vance D.J., Hickey J.M., Joshi S.B., Middaugh C.R., Volkin D.B., Weis D.D., Mantis N.J. (2017). High-definition mapping of four spatially distinct neutralizing epitope clusters on rivax, a candidate ricin toxin subunit vaccine. Clin. Vaccine Immunol..

[12] Pincus S.H., Bhaskaran M., Brey R.N., Didier P.J., Doyle-Meyers L.A., Roy C.J. (2015). Clinical and pathological findings associated with aerosol exposure of macaques to ricin toxin. Toxins (Basel).

[13] Smallshaw J.E., Richardson J.A., Vitetta E.S. (2007). Rivax, a recombinant ricin subunit vaccine, protects mice against ricin delivered by gavage or aerosol. Vaccine.

[14] Roy C.J., Brey R.N., Mantis N.J., Mapes K., Pop I.V., Pop L.M., Ruback S., Killeen S.Z., Doyle-Meyers L., Vinet-Oliphant H.S. (2015). Thermostable ricin vaccine protects rhesus macaques against aerosolized ricin: Epitope-specific neutralizing antibodies correlate with protection. Proc. Natl. Acad. Sci. USA.

[15] Audi J., Belson M., Patel M., Schier J., Osterloh J. (2005). Ricin poisoning: A comprehensive review. JAMA.

[16] Vance D.J., Mantis N.J. (2016). Progress and challenges associated with the development of ricin toxin subunit vaccines. Expert Rev. Vaccines.

[17] Smallshaw J.E., Richardson J.A., Pincus S., Schindler J., Vitetta E.S. (2005). Preclinical toxicity and efficacy testing of rivax, a recombinant protein vaccine against ricin. Vaccine.

[18] O’Hara J.M., Kasten-Jolly J.C., Reynolds C.E., Mantis N.J. (2014). Localization of non-linear neutralizing b cell epitopes on ricin toxin’s enzymatic subunit (rta). Immunol. Lett..

[19] O’Hara J.M., Neal L.M., McCarthy E.A., Kasten-Jolly J.A., Brey R.N., Mantis N.J. (2010). Folding domains within the ricin toxin a subunit as targets of protective antibodies. Vaccine.

[20] Rudolph M.J., Vance D.J., Cassidy M.S., Rong Y., Mantis N.J. (2017). Structural analysis of single domain antibodies bound to a second neutralizing hot spot on ricin toxin’s enzymatic subunit. J. Biol. Chem..

[21] Rudolph M.J., Vance D.J., Cassidy M.S., Rong Y., Shoemaker C.B., Mantis N.J. (2016). Structural analysis of nested neutralizing and non-neutralizing b cell epitopes on ricin toxin’s enzymatic subunit. Proteins.

[22] Rudolph M.J., Vance D.J., Cheung J., Franklin M.C., Burshteyn F., Cassidy M.S., Gary E.N., Herrera C., Shoemaker C.B., Mantis N.J. (2014). Crystal structures of ricin toxin’s enzymatic subunit (rta) in complex with neutralizing and non-neutralizing single-chain antibodies. J. Mol. Biol..

[23] Vance D.J., Tremblay J.M., Rong Y., Angalakurthi S.K., Volkin D.B., Middaugh C.R., Weis D.D., Shoemaker C.B., Mantis N.J. (2017). High-resolution epitope positioning of a large collection of neutralizing and nonneutralizing single-domain antibodies on the enzymatic and binding subunits of ricin toxin. Clin. Vaccine Immunol..

[24] Lemley P.V., Amanatides P., Wright D.C. (1994). Identification and characterization of a monoclonal antibody that neutralizes ricin toxicity in vitro and in vivo. Hybridoma.

[25] Westfall J., Yates J.L., Van Slyke G., Ehrbar D., Measey T., Straube R., Donini O., Mantis N.J. (2018). Thermal stability and epitope integrity of a lyophilized ricin toxin subunit vaccine. Vaccine.

[26] O’Hara J.M., Mantis N.J. (2013). Neutralizing monoclonal antibodies against ricin’s enzymatic subunit interfere with protein disulfide isomerase-mediated reduction of ricin holotoxin in vitro. J. Immunol. Methods.

[27] Van Slyke G., Angalakurthi S.K., Toth R.T., Vance D.J., Rong Y., Ehrbar D., Shi Y., Middaugh C.R., Volkin D.B., Weis D.D. (2018). Fine-specificity epitope analysis identifies contact points on ricin toxin recognized by protective monoclonal antibodies. ImmunoHorizons.

[28] Thomas J.C., O’Hara J.M., Hu L., Gao F.P., Joshi S.B., Volkin D.B., Brey R.N., Fang J., Karanicolas J., Mantis N.J. (2013). Effect of single-point mutations on the stability and immunogenicity of a recombinant ricin a chain subunit vaccine antigen. Hum. Vaccin. Immunother..

[29] Smallshaw J.E., Firan A., Fulmer J.R., Ruback S.L., Ghetie V., Vitetta E.S. (2002). A novel recombinant vaccine which protects mice against ricin intoxication. Vaccine.

[30] Bai Y., Milne J.S., Mayne L., Englander S.W. (1993). Primary structure effects on peptide group hydrogen exchange. Proteins.

[31] Bazzoli A., Vance D.J., Rudolph M.J., Rong Y., Angalakurthi S.K., Toth R.T.t., Middaugh C.R., Volkin D.B., Weis D.D., Karanicolas J. (2017). Using homology modeling to interrogate binding affinity in neutralization of ricin toxin by a family of single domain antibodies. Proteins.

[32] Legler P.M., Brey R.N., Smallshaw J.E., Vitetta E.S., Millard C.B. (2011). Structure of rivax: A recombinant ricin vaccine. Acta Crystallogr. D Biol. Crystallogr..

[33] Rudolph M.J., Vance D.J., Kelow S., Angalakurthi S.K., Nguyen S., Davis S.A., Rong Y., Middaugh C.R., Weis D.D., Dunbrack R. (2018). Contribution of an unusual cdr2 element of a single domain antibody in ricin toxin binding affinity and neutralizing activity. Protein Eng. Des. Sel..

[34] Frankel A., Welsh P., Richardson J., Robertus J.D. (1990). Role of arginine 180 and glutamic acid 177 of ricin toxin a chain in enzymatic inactivation of ribosomes. Mol. Cell. Biol..

[35] Ready M.P., Kim Y., Robertus J.D. (1991). Site-directed mutagenesis of ricin a-chain and implications for the mechanism of action. Proteins.

[36] Wahome P.G., Robertus J.D., Mantis N.J. (2012). Small-molecule inhibitors of ricin and shiga toxins. Curr. Top. Microbiol. Immunol..

[37] Weston S.A., Tucker A.D., Thatcher D.R., Derbyshire D.J., Pauptit R.A. (1994). X-ray structure of recombinant ricin a-chain at 1.8 a resolution. J. Mol. Biol..

[38] Fernandez E., Kose N., Edeling M.A., Adhikari J., Sapparapu G., Lazarte S.M., Nelson C.A., Govero J., Gross M.L., Fremont D.H. (2018). Mouse and human monoclonal antibodies protect against infection by multiple genotypes of japanese encephalitis virus. MBio.

[39] Gribenko A.V., Parris K., Mosyak L., Li S., Handke L., Hawkins J.C., Severina E., Matsuka Y.V., Anderson A.S. (2016). High resolution mapping of bactericidal monoclonal antibody binding epitopes on staphylococcus aureus antigen mntc. PLoS Pathog..

[40] Lim X.X., Chandramohan A., Lim X.E., Crowe J.E., Lok S.M., Anand G.S. (2017). Epitope and paratope mapping reveals temperature-dependent alterations in the dengue-antibody interface. Structure.

[41] Maddaloni M., Cooke C., Wilkinson R., Stout A.V., Eng L., Pincus S.H. (2004). Immunological characteristics associated with the protective efficacy of antibodies to ricin. J. Immunol..

[42] Noy-Porat T., Rosenfeld R., Ariel N., Epstein E., Alcalay R., Zvi A., Kronman C., Ordentlich A., Mazor O. (2016). Isolation of anti-ricin protective antibodies exhibiting high affinity from immunized non-human primates. Toxins (Basel).

[43] Vance D.J., Mantis N.J. (2012). Resolution of two overlapping neutralizing b cell epitopes within a solvent exposed, immunodominant alpha-helix in ricin toxin’s enzymatic subunit. Toxicon.

[44] Vance D.J., Tremblay J.M., Mantis N.J., Shoemaker C.B. (2013). Stepwise engineering of heterodimeric single domain camelid vhh antibodies that passively protect mice from ricin toxin. J. Biol. Chem..

