# Supplementary material for: A Collection of Single-Domain Antibodies that Crowd Ricin Toxin’s Active Site
Source: Antibodies (Basel). 2018 Dec 17;7(4):45. doi: 10.3390/antib7040045 (PMC6374049; doi:10.3390/antib7040045)
Supplement: Supplementary file 1 [file antibodies-07-00045-s001.pdf]

SUPPLEMENTARY TABLES

Supplementary Table 1. RiVax peptic peptides (with indicated peptide number and amino acid residues)

Pep #	start	end	Pep #	start	end	Pep #	start	end	Pep #	start	end
**1**	0	11	**36**	92	103	**71**	162	168	**106**	217	232
**2**	12	20	**37**	92	107	**72**	165	168	**107**	217	240
**3**	12	24	**38**	93	99	**73**	165	171	**108**	218	225
**4**	21	24	**39**	93	107	**74**	168	171	**109**	218	232
**5**	25	32	**40**	102	107	**75**	169	173	**110**	220	232
**6**	25	37	**41**	103	107	**76**	172	181	**111**	221	232
**7**	28	37	**42**	104	107	**77**	175	181	**112**	226	232
**8**	33	59	**43**	104	109	**78**	178	181	**113**	226	240
**9**	37	59	**44**	108	117	**79**	182	186	**114**	227	240
**10**	38	45	**45**	108	118	**80**	182	187	**115**	232	240
**11**	38	55	**46**	108	122	**81**	182	188	**116**	232	243
**12**	38	57	**47**	118	122	**82**	182	190	**117**	232	248
**13**	38	59	**48**	119	126	**83**	182	204	**118**	233	243
**14**	56	59	**49**	123	126	**84**	187	204	**119**	233	244
**15**	58	61	**50**	123	129	**85**	188	204	**120**	233	246
**16**	58	68	**51**	123	133	**86**	189	204	**121**	233	248
**17**	60	68	**52**	123	135	**87**	189	206	**122**	240	243
**18**	60	69	**53**	127	133	**88**	191	204	**123**	241	244
**19**	62	68	**54**	127	135	**89**	191	207	**124**	241	246
**20**	69	72	**55**	130	135	**90**	195	204	**125**	241	248
**21**	69	73	**56**	130	151	**91**	205	210	**126**	243	248
**22**	69	74	**57**	133	144	**92**	205	214	**127**	244	248
**23**	70	74	**58**	134	146	**93**	205	216	**128**	245	248

**24**	72	79	**59**	134	151	**94**	205	217	**129**	247	253
**25**	72	91	**60**	136	146	**95**	207	214	**130**	247	254
**26**	73	79	**61**	136	147	**96**	207	216	**131**	247	255
**27**	73	91	**62**	136	151	**97**	207	217	**132**	249	253
**28**	75	79	**63**	146	150	**98**	208	214	**133**	249	254
**29**	75	91	**64**	147	150	**99**	208	216	**134**	249	255
**30**	80	91	**65**	147	151	**100**	208	217	**135**	255	267
**31**	80	92	**66**	148	151	**101**	211	216	**136**	256	267
**32**	84	91	**67**	152	161	**102**	211	217	**137**	257	267
**33**	92	99	**68**	152	164	**103**	212	216	**138**	258	267
**34**	92	101	**69**	153	164	**104**	217	220			
**35**	92	102	**70**	162	167	**105**	217	225			

**Table S2. HX-MS Analysis of V_HHs in cluster 3.1**			
	**RTA strong and intermediate protected elements[a]**		
**V_HH**	**Peptides**	**Residues**	**Secondary Structure(s)**
V1B11	49-54	123-135	α-helix C
	94-102	205-217	α-helix G
	132-134	249-255	
JNM-D1	55	130-135	α-helix C
	50-54	123-135	α-helix C
	91	205-210	α-helix G
V2A11	49	123-126	α-helix C
	50-53	123-135	α-helix C
	92-103	205-217	α-helix G
	106-116	218-243	β-strands i, j
	132-134	249-255	
V6H8	48-52, 54	119-135	α-helix C
	94,95,97,98,100,102	205-217	α-helix G
	132-134	249-254	
V6A6	48-52, 54	119-135	α-helix C
	91-102	205-217	α-helix G
	130-134	247-255	
V6A7	49	123-126	N-terminus α-helix C
	48, 50-52, 54	119-135	α-helix C

	92-100	205-217	α-helix G
	132-134	247-255	
V6G10	49	123-136	N-term α-helix C
	48,50-52,54	119-135	α-helix C
	92-100	205-217	α-helix G
	132-134	247-255	
V8C7	49	123-126	N-term α-helix C
	48, 50-54	119-135	α -helix C
	91-102	205-217	α-helix G
	132-134	249-255	
V8E6	49	123-126	α-helix C
	47, 50-55	119-135	α -helix C
	92-103	205-217	α-helix G
	129-134	247-255	
V1G6	50-54	123-135	α-helix C
	94,97-98,100,102	205-217	α-helix G
	109-111	218-232	β-strand i
	112-113, 115	226-240	β-strands i, j
	132-134	249-255	
V2G10	47, 50-54	118-135	α-helix C
	55	130-135	C-term α-helix C
	92-103	205-217	α-helix G
	111-113	221-240	β-strands i, j

	132-134	249-255	
V5A2	49-53	123-135	α-helix C
	54,55	127-135	α-helix C
	91	205-210	α-helix G
	114	227-240	β-strands i, j
	112-113,115-116,119	226-243	β-strands i, j
	132-134	249-255	
JIV-F6	54	127-135	α-helix C
	51-53	123-133	α-helix C
	91	205-210	α-helix G
	113-115	226-240	β-strands i, j
	129-131	249-255	
V1B10	54	127-135	α-helix C
	51-53	123-133	α -helix C
	91	205-210	α-helix G
	113,115,116	226-243	β-strands i, j
	132-134	249-255	

[a], Peptides and corresponding amino acid residues on RiVax are indicated in supplementary Table S2.

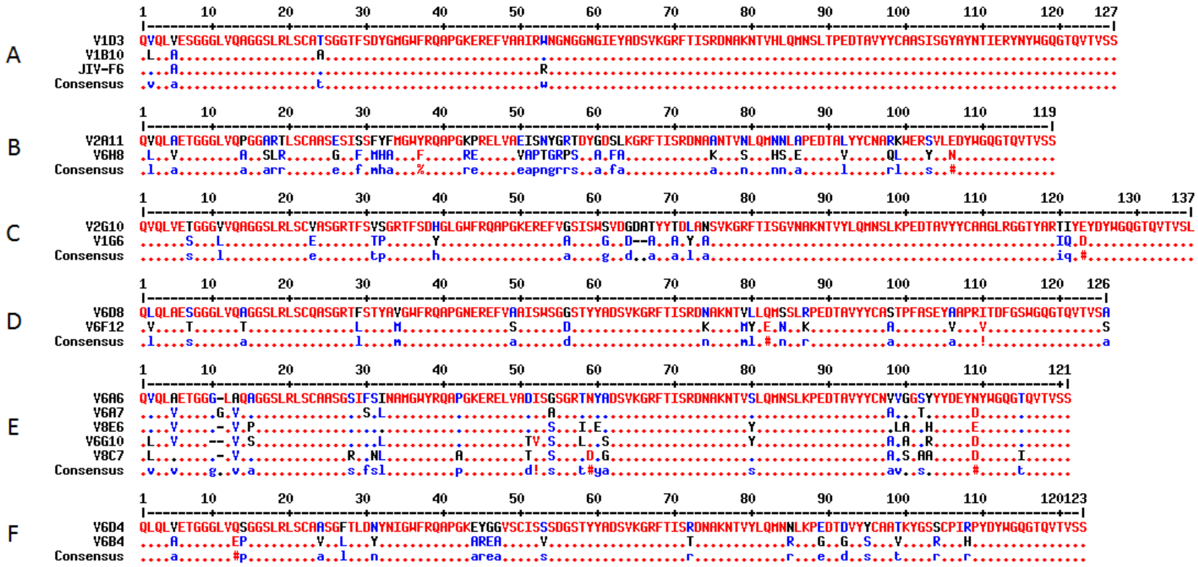

Figure S1. Alignment of cluster 3 V_HH families. The predicted DNA sequences of the V_HHs noted above were aligned using Multalin. The families shown here correspond to those presented in Table 1.

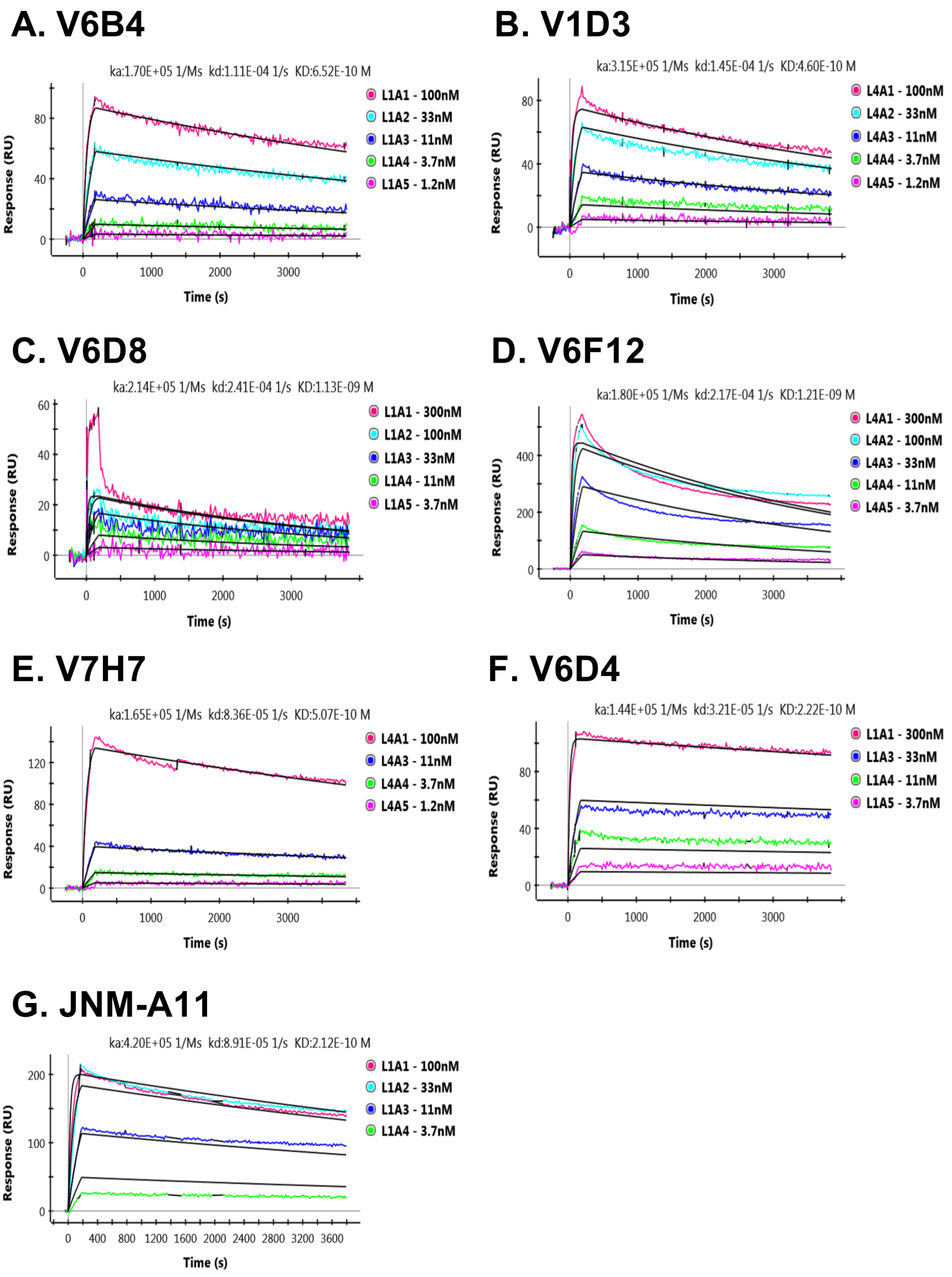

Figure S2. Representative sensorgrams of Cluster 3 V_HHs. Ricin was immobilized on a general layer compact (GLC), as described in Materials and Methods section, then probed with V_HHs (Panels A-G) at indicated molar concentrations (see legends). All kinetic experiments were performed at 25°C. Kinetic constants for the antibody/ricin interactions were obtained with ProteOn Manager software 3.1.0 (Bio-Rad Inc.) using the Langmuir fit model.

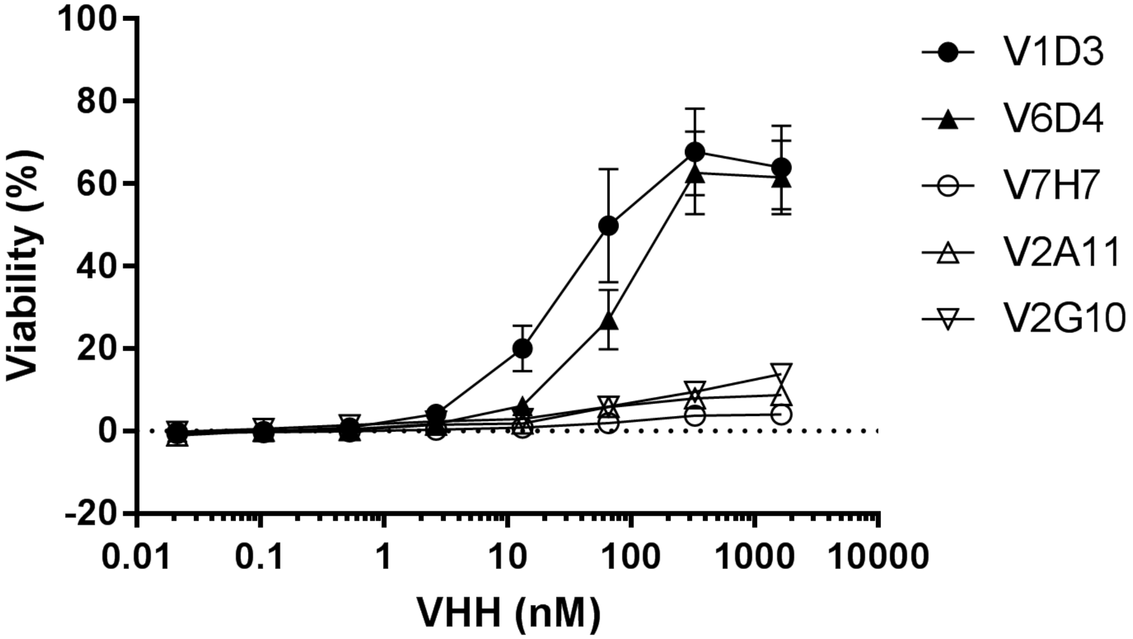

Figure S3. Representative toxin-neutralizing activities of cluster 3 V_HHs. The indicated V_HHs were mixed with ricin (10 ng/ml) and then applied to Vero cells, as described in the Materials and Methods. Cell viability was measured ~48 h later. Among the 21 cluster 3 V_HHs, only V1D3 and V6D4 demonstrated significant (albeit moderate to weak) toxin-neutralizing activity.

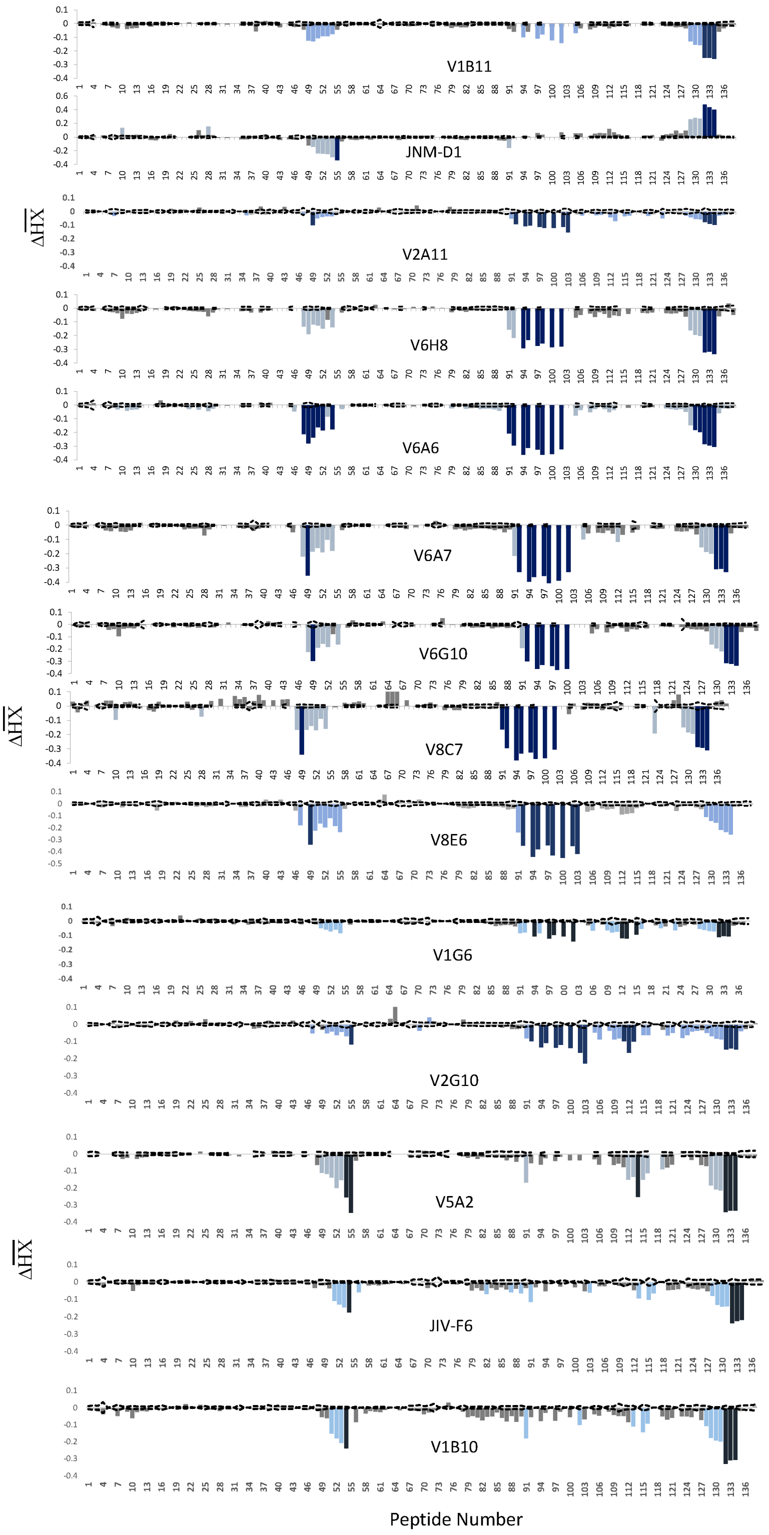

Figure S4. HX-MS analysis of RiVax bound to V_HHs in subcluster 3.1. The $\Delta\overline{\mathrm{HX}}$ values for each RiVax peptide are shown for V_HHs denoted in the figure. The $\Delta\overline{\mathrm{HX}}$ values are clustered using k-means clustering into three categories: strong (deep blue), intermediate (light blue) or no significant protection (gray). The dotted lines represent "3σ" confidence intervals for statistically significant changes in hydrogen exchange.

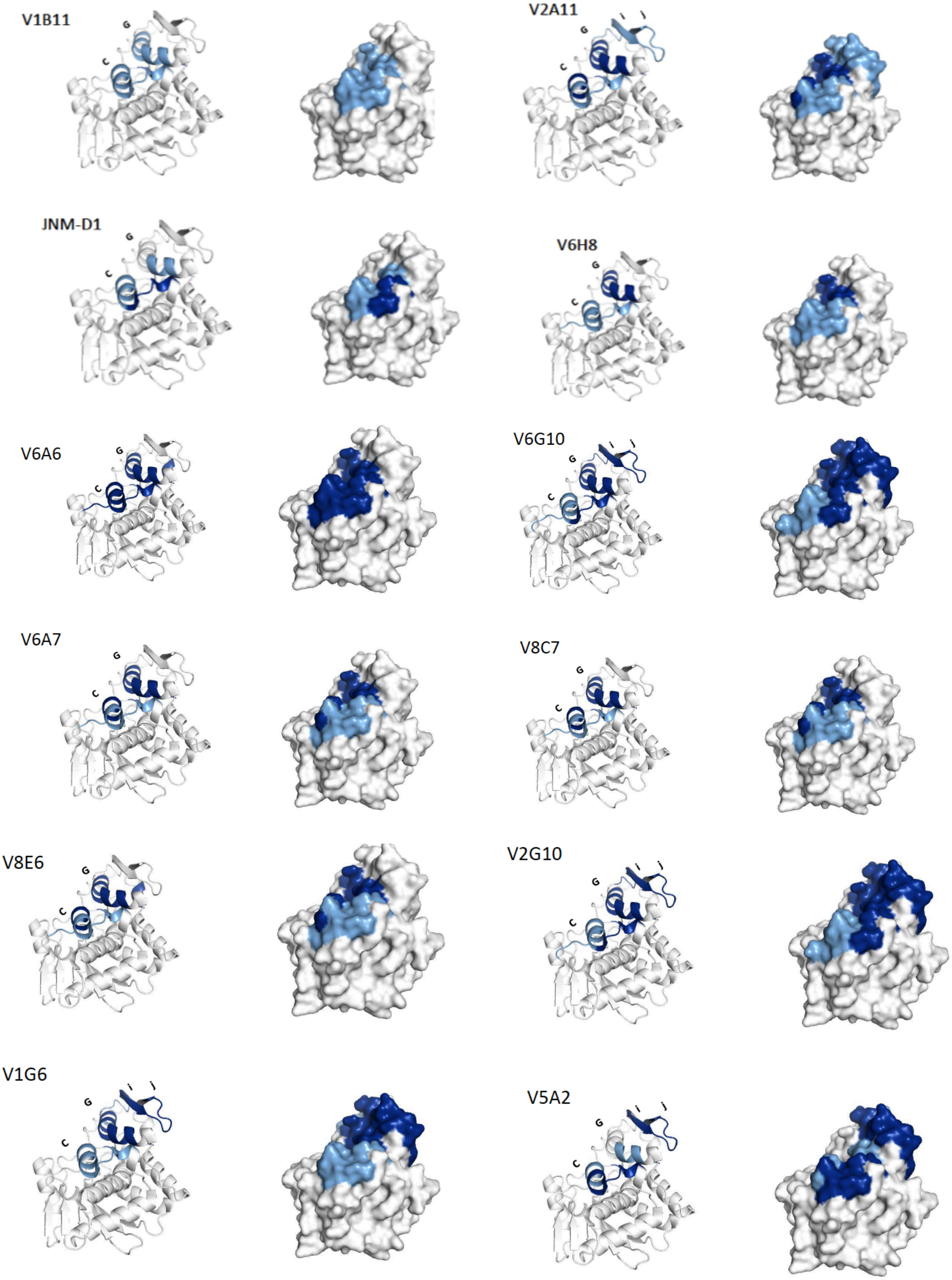
V1B11
V2A11
JNM-D1
V6H8
V6A6
V6G10
V6A7
V8C7
V8E6
V2G10
V1G6
V5A2

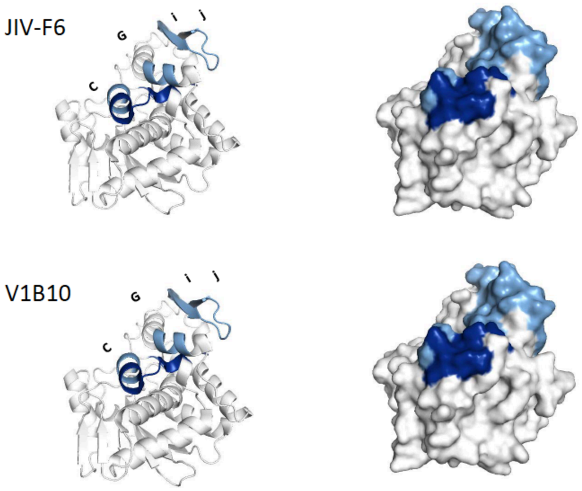

Figure S5. Epitope localization of subcluster 3.1 V_HHs on the surface of RiVax. The HX protection categories shown in Figure S4 were mapped onto the crystal structure of RiVax indicated V_HHs. The most relevant secondary structure elements are labelled. The color shading corresponds to strong (deep blue), intermediate (light blue) or no significant protection (gray), as represented in Figure S4.
